# Supplementary material for: Exploring the influence of pore shape on conductance and permeation
Source: Biophys J. 2024 Jul 6;123(18):3107–19. doi: 10.1016/j.bpj.2024.07.010 (PMC11427812; doi:10.1016/j.bpj.2024.07.010)
Supplement: Document S1. Figures S1 and S2 [file mmc1.pdf]

Biophysical Journal, Volume *123*

Supplemental information

Exploring the influence of pore shape on conductance and permeation

David Seiferth and Philip C. Biggin

SUPPLEMENTAL INFORMATION

Exploring the Influence of Pore Shape on Conductance and Permeation

David Seiferth[1,2] *and Philip C. Biggin*[2*]

[1] Clarendon Laboratory, Department of Physics, University of Oxford, Oxford, OX1 3PU, UK

[2] Structural Bioinformatics and Computational Biochemistry, Department of Biochemistry, University of Oxford, Oxford, OX1 3QU, UK

* To whom correspondence should be addressed:

philip.biggin@bioch.ox.ac.uk

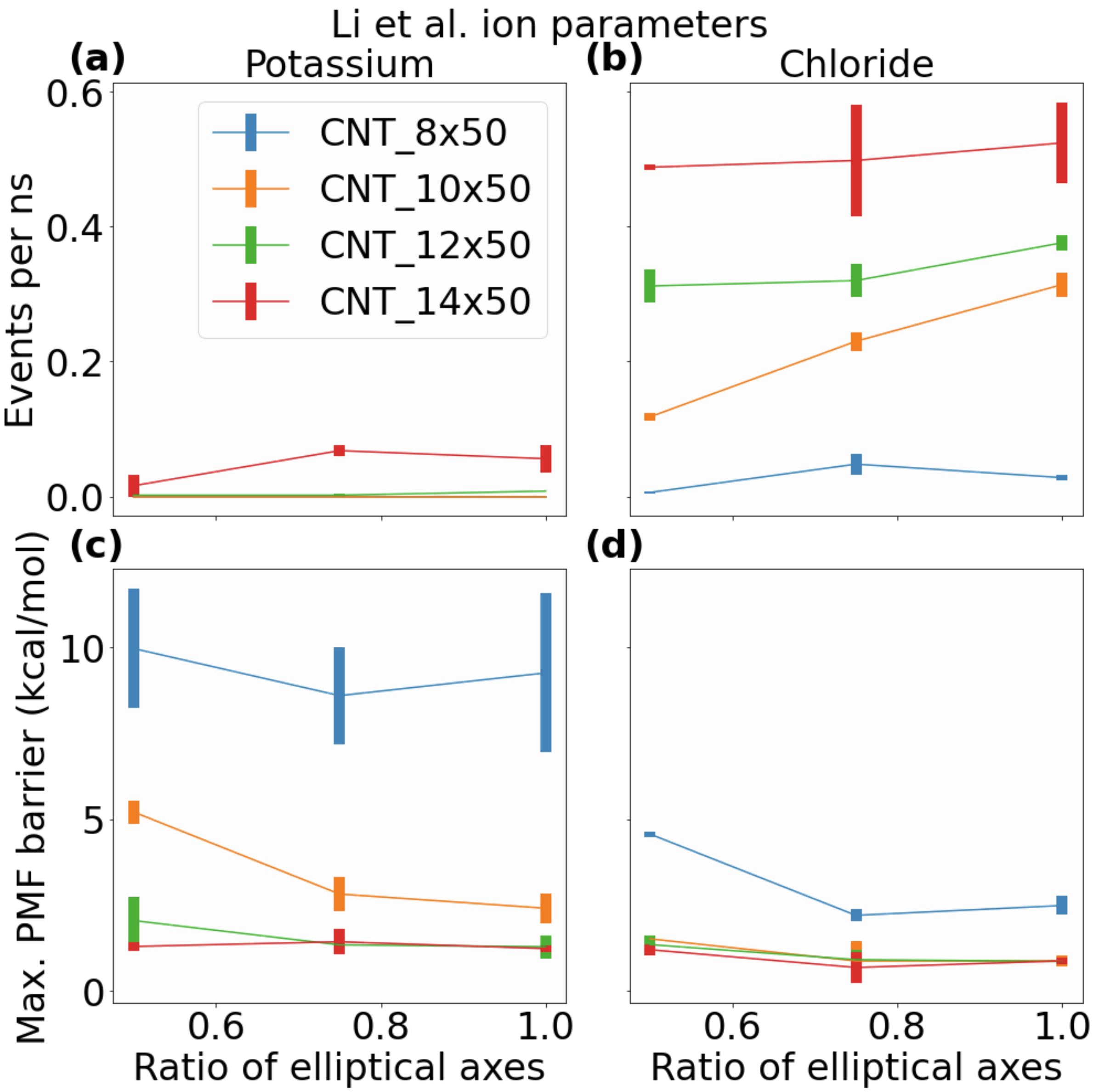

Figure S1. *Barrier for ion permeation and ion conductance for different carbon nanotubes (CNTs) as a function of the ratio between of the elliptic radii using ion parameters from Li et al (2015). The conductance is measured as events per ns observed in multiple 250 ns repeats with external potential of 500 mV for potassium* ***(a)*** *and chloride* ***(b)*** *ions. The energetic barrier is the maximum of the Potential of Mean Force (PMF) obtained from umbrella sampling and is computed for CNTs with potassium* ***(c)*** *and chloride* ***(d)*** *ions. The different CNT systems 8 x 50, 10 x 50, 12 x 50, and 14 x 50 (see Table 1 for corresponding HOLE radii) are all 50 Å in length and shown orange, green and red lines, respectively.*

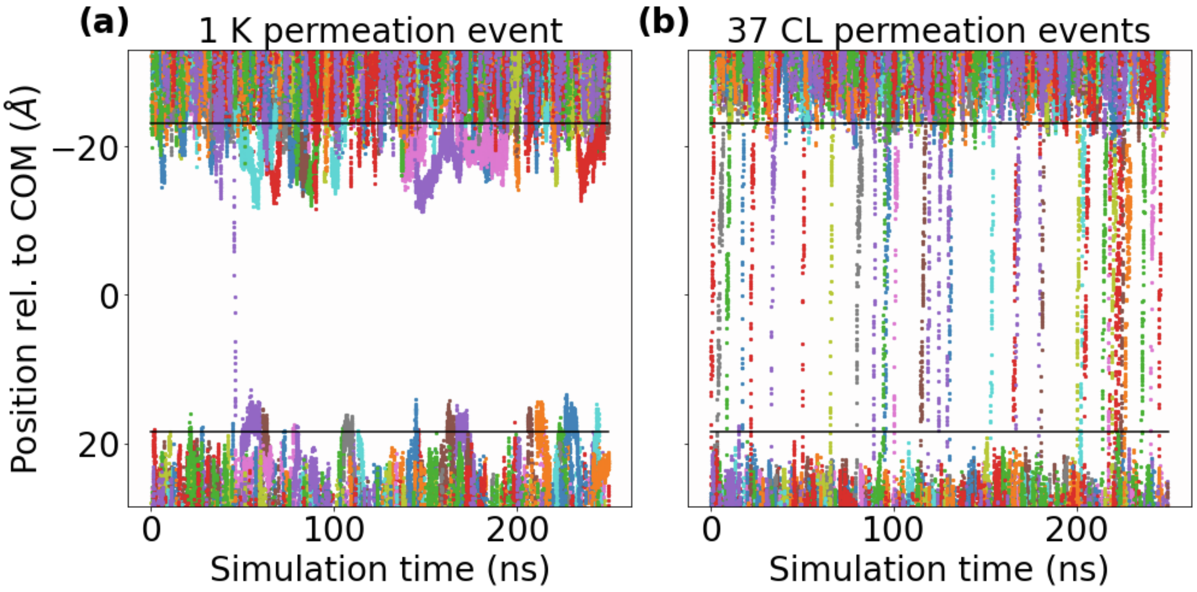

Figure S2. *Time series of the positions of permeating ions. Every colour corresponds to a different potassium (a) or chloride (b) ion. The black lines represent the position of the phosphate atoms of the POPC bilayer. The z-coordinate of the ions is plotted in a coordinate frame where the centre of mass (COM) of the 12x50 CNT system with ratio ½ is the origin.*
