# Supplementary material for: Exploring the influence of pore shape on conductance and permeation
Source: Biophys J. 2024 Jul 6;123(18):3107–19. doi: 10.1016/j.bpj.2024.07.010 (PMC11427812; doi:10.1016/j.bpj.2024.07.010)
Supplement: Document S2. Article plus supporting material [file mmc2.pdf]

Biophysical Journal
Article

Biophysical Society

Exploring the influence of pore shape on conductance and permeation

David Seiferth[1,2] and Philip C. Biggin[2,*]
[1]Clarendon Laboratory, Department of Physics, University of Oxford, Oxford, United Kingdom and [2]Structural Bioinformatics and Computational Biochemistry, Department of Biochemistry, University of Oxford, Oxford, United Kingdom

ABSTRACT There are increasing numbers of ion channel structures featuring heteromeric subunit assembly, exemplified by synaptic $\alpha 1\beta_B$ glycine and $\alpha 4\beta 2$ nicotinic receptors. These structures exhibit inherent pore asymmetry, but the relevance of this to function is unknown. Furthermore, molecular dynamics simulations performed on symmetrical homomeric channels often lead to thermal distortion whereby conformations of the resulting ensemble are also asymmetrical. When functionally annotating ion channels, researchers often rely on minimal constrictions determined via radius-profile calculations performed with computer programs, such as HOLE or CHAP, coupled with an assessment of pore hydrophobicity. However, such tools typically employ spherical probe particles, limiting their ability to accurately capture pore asymmetry. Here, we introduce an algorithm that employs ellipsoidal probe particles, enabling a more comprehensive representation of the pore geometry. Our analysis reveals that the use of nonspherical ellipsoids for pore characterization provides a more accurate and easily interpretable depiction of conductance. To quantify the implications of pore asymmetry on conductance, we systematically investigated carbon nanotubes with varying degrees of pore asymmetry as model systems. The conductance through these channels shows surprising effects that would otherwise not be predicted with spherical probes. The results have broad implications not only for the functional annotation of biological ion channels but also for the design of synthetic channel systems for use in areas such as water filtration. Furthermore, we make use of the more accurate characterization of channel pores to refine a physical conductance model to obtain a heuristic estimate for single-channel conductance. The code is freely available, obtainable as pip-installable python package and provided as a web service.

SIGNIFICANCE Ion channels composed of different subunits possess an inherent asymmetry, as has been confirmed structurally by recent cryo-EM structures. Characterization of the pore-geometry is an important step in the functional annotation of ion channels and typically performed using a spherical probe methodology such as that used by HOLE. Such an approach provides an intuitive radial metric that describes the constrictions of the pore. However, for asymmetrical pores, it will have reduced accuracy. Here, we present an easy-to-use ellipsoidal approach that systematically examines the influence of asymmetry on conductance in carbon-nanotube model systems and recent ion channels. Our results show surprising effects that would otherwise not be predicted with spherical probes. We suggest this approach may be useful for future ion channel annotation.

INTRODUCTION

Advances in structural biology, coupled with the emergence of artificial intelligence-driven structure prediction algorithms, have significantly expanded our knowledge of three-dimensional ion channel and nanopore structures in various conformational states. Notably, AlphaFold (1) and cryoelectron microscopy (cryo-EM) have played a pivotal role in unraveling the intricate details of these molecular structures. However, despite these remarkable strides, the functional annotation of these structures remains a critical challenge. To address this gap, several tools have been developed, such as HOLE (2), Caver (3), MOLEonline (4), and the Channel Annotation Package (CHAP) (5), which enable the analysis of the physical dimensions and characteristics of the pores running through ion channels.

One of the first programs to compute the pore radius of an ion channel was HOLE (2). The HOLE program utilizes a Monte Carlo simulated annealing procedure to determine the optimal route for a sphere with varying radius to traverse

Submitted April 24, 2024, and accepted for publication July 5, 2024.
*Correspondence: philip.biggin@bioch.ox.ac.uk
Editor: Grace Brannigan.
https://doi.org/10.1016/j.bpj.2024.07.010
© 2024 The Author(s). Published by Elsevier Inc. on behalf of Biophysical Society.
This is an open access article under the CC BY license (http://creativecommons.org/licenses/by/4.0/).

through the channel. On the other hand, CHAP (5), combines calculations of pore radius, hydrophobicity, and water density to predict hydrophobic gates in ion channels. CHAP is written in C++ and utilizes the trajectory analysis framework of the MD simulation software GROMACS.

Other tools, such as MOLEonline (4) and CAVER (3), have also been employed for the detection and characterization of channels, pores, and tunnels within biomacromolecules and do not use a probe-based algorithm for path finding. MOLEonline provides information about channel-lining residues, physicochemical properties, and identifies cavities using Voronoi diagrams and molecular surfaces. CAVER employs a grid-based approach and convex approximation to determine the lowest-cost centerline path between a given starting point and the molecule's surface (6). In later versions, CAVER also uses Voronoi diagrams (7). The visualization of the CAVER or MOLEonline path, despite not being probe based in its algorithm, relies on the utilization of probe particles. By utilizing the maximum radii for each node along the path, researchers can effectively visualize and analyze the characteristics of the identified channels or pores. Although some methods offer the chance to visualize detailed (highly asymmetric) surfaces, probe-based methods offer a more intuitive way to interpret the geometry in terms of simple statistics like radii.

The HOLE program, in particular, has been commonly used by structural biologists as a way to quickly (and robustly) provide key information on pore geometry. Until recently, the vast majority of transmembrane channel structures were highly symmetrical, usually as a direct consequence of homomeric subunits forming higher-order multimer structures coupled with symmetrical averaging techniques in the structural processing. Homomeric channels are not perfectly symmetric in a continuous rotational sense, as they are only symmetric about an n-fold rotational axis. However, as structural biology techniques improve, the number of structures that exhibit inherent asymmetry is increasing and understanding its potential role and relationship to function is gaining attention. Minniberger et al. (8) explored the asymmetry and ion selectivity properties of bacterial NaK channel mutants derived from ionotropic glutamate receptors. They observed structural asymmetry in the upper part of the selectivity filter, proposing that local asymmetrical conformational changes could efficiently alter ion channel function without requiring large-scale conformational transitions. Similarly, Roy et al. (9) studied a nonselective sodium-potassium ion channel with structural asymmetry in the selectivity filter, suggesting that different degrees of conformational plasticity are required for conducting potassium and sodium.

Zhang et al. (10) proposed a model for asymmetric activation of the 5-HT$_3$A serotonin receptor, while Shi et al. (11) highlighted the permeation mechanisms of sodium (Na^+) and potassium (K^+) in NaK channels. Lewis et al. (12) observed structural changes between Na^+- and K^+-bound states, accompanied by increased structural heterogeneity with Na^+. Chugunov et al. (13) reported asymmetric gating at the selectivity filter in molecular dynamics (MD) simulations of the TRPV1 pore upper gate. These studies collectively emphasize that conformational plasticity and structural asymmetry in the selectivity filter play crucial roles in regulating ion selectivity and conductance. Furthermore, local asymmetrical conformational changes emerge as an energetically efficient means to modulate ion channel function without requiring global conformational transitions across all subunits.

The presence of asymmetry in ion channel pores brings into question as to whether the use of a spherical probe is appropriate. In fact, this was anticipated many years ago, and the HOLE software already provides a so-called CAPSULE option to measure pore anisotropy with a spherocylinder. However, an asymmetrical pore can be more accurately described with an ellipsoidal probe. Along the channel axis, two radii are reported corresponding to the longer and shorter axes of the ellipsoid. The volume of the pore characterized with ellipsoidal probe particles will be larger than the volume found when using spherical probe particles. To that end and building upon the well-established HOLE program and its MDAnalysis Python API (14), we have developed a program that we call PoreAnalyser. It is available as a pip-installable python package (https://github.com/bigginlab/PoreAnalyser) and interactive web service (https://poreanalyser.bioch.ox.ac.uk) and allows users to capture asymmetry via the use of an ellipsoidal probe. To demonstrate its use, we report here an assessment of the influence of asymmetry on conductance predictions. It is worth noting that, even with an ellipsoidal probe particle, the asymmetry of a channel cannot always be accounted for, as the ellipsoidal shape is often still more symmetric than the channel structure. However, reporting two radii is a good compromise to provide an easily interpretable representation of the pore geometry.

To systematically quantify the implications of pore asymmetry, we investigated carbon nanotubes (CNTs) as model systems, exploring the conductance through channels with varying degrees of pore asymmetry. The investigation builds upon the cylindrical approximation model, a fundamental physical model employed to predict channel conductance. Hille (15) characterized homogeneous conducting materials by a bulk property known as resistivity ρ_{bulk}, representing the inverse of conductivity κ_{bulk}. The conductance model, treating the channel as a cylinder, considered both pore resistance R_{pore} associated with the length of the pore and the so-called access resistance at the pore entrance (R_{access}) and as in Eq. 1.

$$R = R_{pore} + R_{access} = \rho_{bulk}\frac{L}{A} + R_{access} \tag{1}$$

where A denotes the cross-sectional area and L the length of the cylinder. Subsequent studies, such as that by Smart et al.

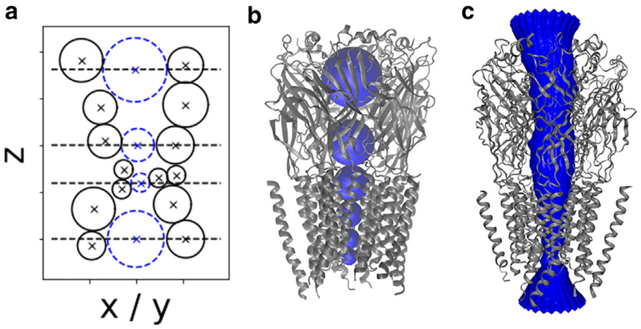

FIGURE 1 Pore geometry calculations with spherical probe particles used in HOLE (2) or CHAP (5). (*a*) The position of a spherical probe (*blue* and *dashed*) is iteratively adjusted in parallel planes to achieve the maximum radius while ensuring there is no overlap with the van der Waals sphere of any atom (*black circles*) within the ion channel. (*b*) Positions of a few probe particles (*blue*) along the pathway of the pore within the $\alpha1\beta_B$ glycine receptor (28) (PDB: 7tvi). (*c*) Surface representation of the pore of the $\alpha1\beta_B$ glycine receptor.

(16), utilized Hille's cylindrical model but neglected access resistance, approximating an ion channel as a series of cylinders with different radii as determined by HOLE analysis. Mejri et al. (17) extended this work using CNTs by revealing that increasing the CNT diameter enhanced conductance values, while an increase in tube length decreased conductance, suggesting a series-like action of access resistance and pore resistance. Manghi et al. (18) further investigated the relative contributions of channel and access resistances in experimentally measured currents.

CNTs are established model systems for ion channels in the realm of MD studies (19,20). Their controllable dimensions make them invaluable tools for investigating ion transport phenomena. CNTs have been explored in the context of dewetting processes within confined hydrophobic regions of channels. Confined hydrophobic regions in ion channels and CNTs may undergo transitions between wet and dry states to gate the pore closed without physical constriction of the permeation pathway—a phenomenon called hydrophobic gating (21). Beckstein et al. (22) contributed to the understanding of dewetting mechanisms by proposing a hydrophobic gating mechanism for nanopores, emphasizing the role of nanopore geometry, diameter, and the hydrophobicity of pore lining residues. In the context of this study, we focused on channels wide enough to rule out dewetting transitions—we only consider conductive CNTs with radii larger than 4 Å whose pores are consistently wetted.

Mendonça et al. (23,24) delved into the intricacies of water diffusion within deformed CNTs characterized by varying degrees of eccentricity. Their investigations provided insights into the behavior of water molecules within these nanoscale conduits, particularly emphasizing the freezing of water under certain conditions. Rezaee et al. (25) studied monoatomic fluid flow through elliptical carbon nanotubes, systematically examining the influence of tube size, temperature, and pressure gradient on the fluid dynamics. Balme et al. (26) contributed to the understanding of ionic transport through hydrophobic nanopores, observing modifications in the ion solvation structure when nanopore diameters were less than 2 nm and varying NaCl concentrations. In addition, Mejri et al. (17) focused on the role of water models (SPC/E, TIP3P, TIP4P/2005) in the study of ionic conductance using MD simulations. Their findings indicated that the choice of water model had no significant impact on the conductance trend concerning increasing tube diameter; however, it did influence conductance values. Notably, hydrogen bond analyses revealed similar water arrangements within the pore for both the SPC/E and TIP4P/2005 models, highlighting the robustness of certain popular water models in capturing essential aspects of ion transport through CNTs. Mejri et al. studied the impact of different chemical CNT functionalization and geometric parameters such as radius and chirality on conductance through MD simulations with an external potential (27). Together, these studies contribute to an understanding of the complex interplay between CNT characteristics, water behavior, and ionic conductance.

We thus chose CNTs as models systems where we can easily control the extent of pore asymmetry in the system. The conductance through these CNTs show surprising effects that would otherwise not be predicted with spherical probes. Building on the cylindrical approximation, we propose an improved physical model to predict conductance. The results have broad implications not only for the functional annotation of biological ion channels, but also for the design of synthetic channel systems for use in areas such as water filtration. We anticipate that PoreAnalyser will play an increasingly important role as the number of asymmetric channel structures increases.

METHODS

Pore geometry calculations

We align the principal axis of the input structure to the z axis by default, although this can be modified as per user preference (users of the web interface can simply upload their own coordinates). To calculate the pore geometry, we employ probe-based pathway finding using spherical probe particles as a first step and then we transform each sphere into an ellipsoid. Initially, HOLE is executed using a spherical probe particle, with the center of mass of the input structure as the default starting point. The position of the probe is optimized in subsequent parallel xy planes to maximize its radius without overlapping the van der Waals sphere of any ion channel atom. Fig. 1 illustrates the schematic representation of van der Waals spheres, the positions of their centers in the xz plane of a channel aligned to the z axis, and a surface representation of the pore. HOLE is integrated into our Python workflow with its MDAnalysis Python API (14).

To incorporate an ellipsoidal probe particle, we iteratively transform the spherical probe particles into ellipsoids. To initiate the calculation, we load the output file from HOLE containing the positions and radii of the probes. Then, we perform a loop through all the spherical probe particles and initialize an ellipsoid using the parameters obtained from the HOLE output. The parameters of the ellipsoid include the position of its center $[x, y, z]$, orientation θ, and radii $[a, b]$, where $a \geq b$. The smaller radius, b, remains constant along the z-coordinate. To optimize the parameters, we employ a Nelder-Mead four-dimensional optimization algorithm, first using smaller

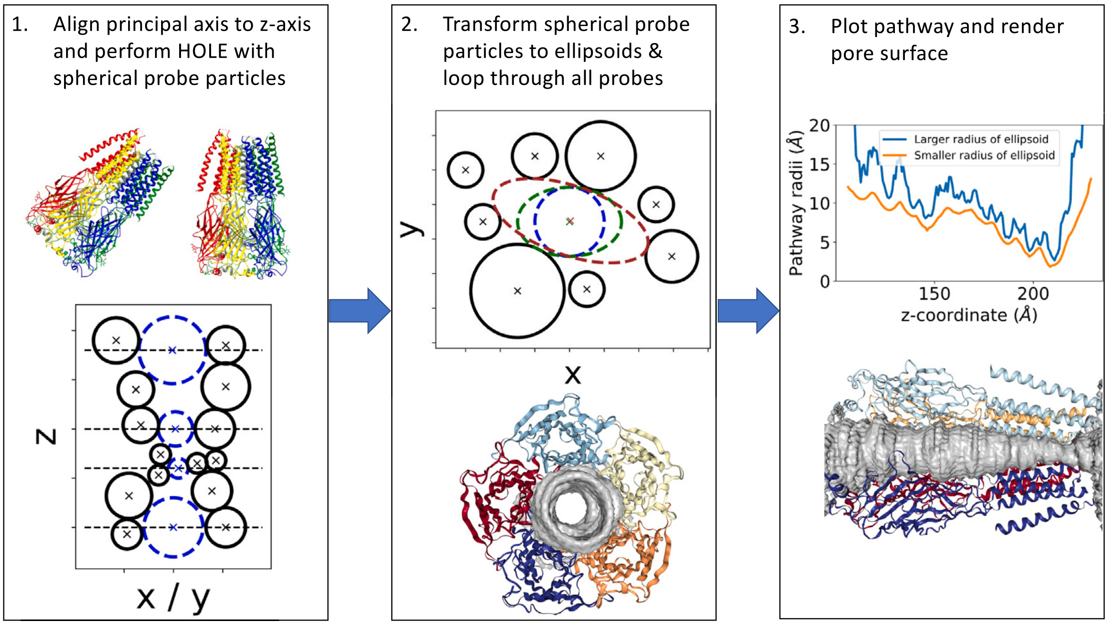

FIGURE 2 Workflow diagram of the web service and the python package. The input protein is aligned to the z axis and then a HOLE analysis is performed (1). The HOLE spheres are then iteratively transformed into ellipsoid (2). As a final step, the pathway is plotted in terms of both the larger and smaller ellipsoid radii and the pore surface visualized (3).

bounds for the parameters [x, y, a, θ], and then with larger boundaries to further increase the ellipsoid (Fig. 2). Finally, we compare the volumes of the pores based on the use of spherical and ellipsoidal probe particles. This comparison provides insights into the impact of probe shape on pore geometry calculations.

Model systems

We employ pristine armchair CNTs capped with hydrogen atoms as a model system, featuring a length of 50 Å, a dimension that enables them to span a lipid bilayer completely. The key characteristic of the CNTs lies in the variation of their radius, carefully selected to be wide enough to prevent dewetting of the pore, ensuring stable hydration throughout the simulation duration. All CNT atoms have zero partial charge. The simulation setup involves the insertion of the CNT into a bilayer (see Fig. 3) composed of 128 1-palmitoyl-2-oleoyl-*sn*-glycero-3-phosphocholine (POPC) lipids, facilitated by the InflateGro method (29). The system is solvated with a 0.15 M KCl solution. Atomistic MD simulations are conducted using GROMACS (30), wherein the CNT is modeled using the OPLS all-atom force field (31), united-atom lipids, and the SPC/E water model (32). Simulation parameters include a time integration leap-frog with a 2 fs time step, constraints applied to bonds using the LINCS (33,34) algorithm, and the particle mesh Ewald method (35,36) employed for handling long-range electrostatic interactions. The temperature is maintained at 300 K. To preserve orientation, the z-coordinates of the CNT are restrained, with harmonic restraints of 500 kJ/mol/nm^2. Introducing varying degrees of asymmetry, we manipulate the area in the xy plane $A_{xy} = \pi r^2 = \pi\, a\, b$, where r represents the radius of the CNT circular plane, a denotes the larger and b the smaller radius of the ellipsoid. The asymmetry is quantified by the ratio of the smaller to the larger elliptic radius (½, ¾, and 1), corresponding to different degrees of eccentricity $e = \sqrt{1 - \frac{b^2}{a^2}}$, depicted in Fig. 3, *a–c*. The cross-sectional area A_{xy} is preserved for varying degrees of eccentricity. To retain a noncircular shape, harmonic restraints are defined such that a major axis of $2a$ and a minor axis of $2b$ are enforced. Notably, the pores remain stably hydrated throughout the entirety of the simulations, providing a robust foundation for investigating the influence of varying degrees of asymmetry on the behavior of CNTs within lipid bilayers. We study (8,8), (10,10), (12,12), (14,14), (16,16), and (18,18) pristine armchair CNTs with different degrees of eccentricity, where the native (8,8) and the (14,14) have HOLE radii of 3.4 and 7.0 Å, respectively (see Table 1).

We denote a (14,14) armchair CNT with length 50 Å as 14 × 50 in this article. The CNT type, denoted by the pair of integers (n,m), signifies that a specific CNT can be derived by rolling a graphene sheet characterized by lattice vectors $\vec{a}_1$ and $\vec{a}_2$. In this configuration, atoms align theoretically atop one another when positioned at a distance of $n\vec{a}_1 + m\vec{a}_2$ on the graphene sheet.

Ion conduction was measured in 250 ns simulations at 150 mM KCl concentration and in the presence of a +500 and −500 mV transmembrane potential difference. This was applied by imposing an external, uniform electric field in the membrane normal direction. Umbrella sampling was performed to obtain one-dimensional single-ion potential of mean force profiles for potassium and chloride passing through the pore of different CNTs with varying degree of asymmetry. Simulation protocols were similar to those detailed above for equilibrium MD simulations. The distance between the respective ion and the center of mass of the CNT structure along the z axis of the simulation box was chosen as the collective variable. Starting configurations were generated by swapping positions of the respective ion with water molecules along the permeation pathway. The minimum of the biasing potential was shifted by 0.2 nm between each umbrella sampling window. A force constant of 500 kJ/mol/nm^2 restrained the z-coordinate of sodium to the respective distance to the center of mass of the protein. In addition to the distance restraint of the reaction coordinate, we introduced flat bottom restraints in the xy plane to force the ion to stay within a cylinder with a sufficiently large radius $r_{cyl} > \max\,(a, b)$, where a and b denote the radii of the elliptical cross-sectional area. Within the cylinder, the ion can move freely, while outside of the cylinder there are high harmonic restraints of 5000 kJ/mol/nm^2, that force the ion back into the cylinder. This cylinder ensures that the ion cannot move laterally away from the CNT when its z-coordinate is restrained to the entry or exit of the CNT. We

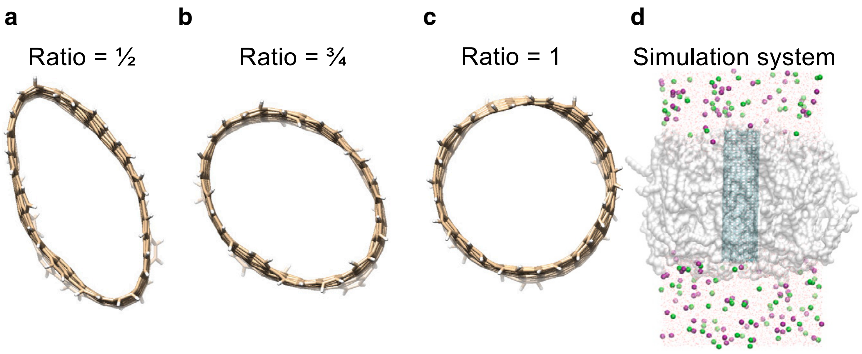

FIGURE 3 Cylindrical carbon nanotubes with varying degrees of asymmetry. View of the xy plane. (a) Elliptical xy plane with a ratio of ½ between smaller and larger radius. (b) Elliptical xy plane with ratio of $^3/_4$ between smaller and larger radius. (c) Circular xy plane (smaller and larger radius of ellipse are identical). The area $A_{xy} = \pi ab$ in the xy plane is conserved and virtual bonds are defined to maintain the degree of asymmetry. (d) Cartoon of the simulation box with the carbon nanotube in cyan, the lipid in gray translucent spheres, water in red and white wire, and K^+ and Cl^- ions in purple and green, respectively.

corrected the energetic barrier as described by Seiferth et al. (37). Unbiasing was performed with the weighted histogram analysis method using the GROMACS implementation (38).

RESULTS AND DISCUSSION

Case studies of biological ion channels

To demonstrate how the geometrical profile of the pore is influenced by the use of an ellipsoidal probe, we investigated the pore geometry of the full-length zebrafish α1β glycine receptor (GlyR) as a case study. GlyR plays a crucial role in inhibitory neuronal signaling in the spinal cord and brainstem. Synaptic GlyRs are a heteromeric assembly of α and β subunits and hence asymmetric (28). We analyzed a cryo-EM structure of the full-length zebrafish α1βGlyR in glycine-bound state (PDB: 7TVI) (28). Our analysis, as depicted in Fig. 4, reveals that the local minima of the pathway radii obtained using a spherical probe particle, representing the smaller radius, coincided with the local minima obtained from the larger radius of the ellipsoid. This observation indicates the consistency and reliability of our calculations. We also plotted the radius profiles from MOLE (4) and CHAP (5) in Fig. 4 *a* and noted that the profiles coincide with the profile obtained from using a spherical probe particle. CHAP does not use the z-coordinate but a custom pore coordinate, s, and hence the CHAP profile differs slightly from MOLE and HOLE using the z-coordinate. Moreover, we found that the ratio of pore volume based on the ellipsoidal and spherical probe particles was approximately 1.6. This indicates that using a spherical probe particle alone would lead to a significant underestimation of the actual pore volume. The incorporation of an ellipsoidal probe particle provides a more accurate representation of the true pore volume, offering valuable insights into the size and dimensions of the GlyR pore. Minniberger et al. (8) observed structural asymmetry of the selectivity filter in x-ray structures of bacterial NaK channel mutants designed to mimic ionotropic glutamate receptors and are nonselective tetrameric cation channels. In Fig. 4 *c*, the radius profile obtained with an ellipsoidal probe particle reveals a high degree of asymmetry in the upper part of the selectivity filter where the larger elliptical axis is twice as long as the smaller one. These findings highlight the importance of considering different probe shapes when calculating pore geometry and emphasize the limitations of relying solely on spherical probe particles for accurate measurements.

TABLE 1 CNTs of length 50 Å and varying HOLE radii

Armchair CNT of type (n,m)	(8,8)	(10,10)	(12,12)	(14,14)	(16,16)	(18,18)
HOLE radius (Å)	3.4	4.7	5.9	7.0	8.3	9.8

Comparison with HOLE capsule option

As mentioned earlier, Smart et al. (16) introduced the CAPSULE option within the HOLE algorithm to allow the user to capture pore asymmetry, a feature that has yet to gain widespread adoption within the scientific community. Departing from the traditional approach of optimizing a single sphere, HOLE with the CAPSULE option extends its capability to a spherocylinder, essentially an extension of a sphere. The spherocylinder is uniquely characterized by two independently moving centers, described by three properties: its radius r and the positions of these two centers with distance L between them (16). Central to the algorithm is the measurement of anisotropy, achieved by ensuring that the axis of the capsule aligns perpendicular to the channel direction vector. Unlike the original routine, which optimized the sphere's radius, the CAPSULE option focuses on maximizing the area of the capsule on a given plane. Subsequently, this area is converted into an effective radius $r_{eff} = \sqrt{r^2 + \frac{L\,r}{\pi}}$, providing a parameter for ease of comparisons. In Fig. 5, we compare the performance of the HOLE CAPSULE algorithm with our PoreAnalyser package, where ellipsoidal probe particles are employed to comprehensively explore the intricacies of pore geometry, providing valuable insights into the relative strengths and limitations of these two distinct algorithms. The smaller elliptical radius b of the PoreAnalyser algorithm and the spherical radius r of HOLE are identical by construction

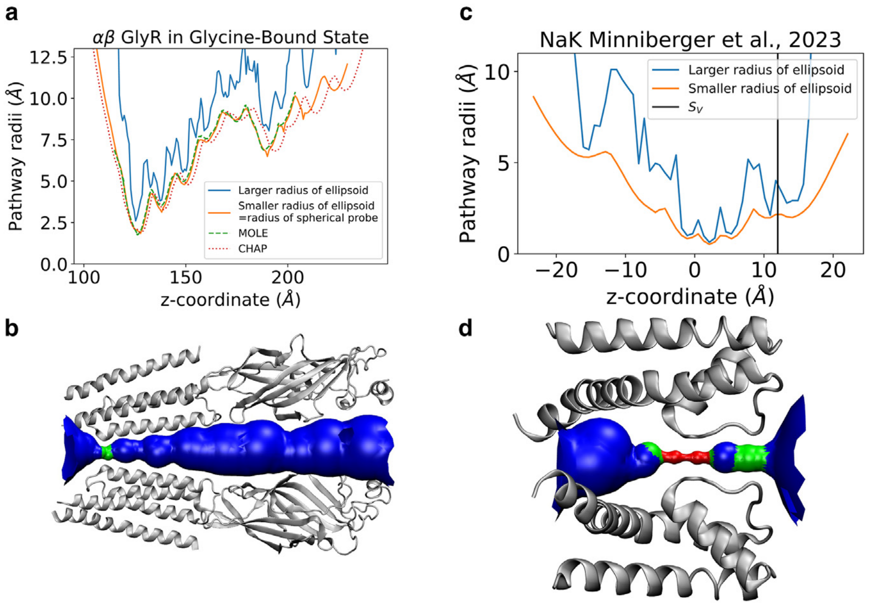

FIGURE 4 Case studies of the $\alpha1\beta_B$ GlyR and NaK channels. (*a*) Pathway analysis for a cryo-EM structure of full-length zebrafish $\alpha1\beta_B$ GlyR with heteromeric subunit assembly in the presence of glycine (28) with the larger and smaller ellipsoid radii shown as blue and orange lines, respectively. The radii profiles for MOLE (4) and CHAP (5) are plotted as green dashed and dotted pink lines, respectively. (*b*) Cartoon showing the pore profile for $\alpha1\beta_B$ (PDB: 7tvi). (*c*) Pathway profile of a of bacterial channel NaK mutant (PDB: 7oph: larger and smaller ellipsoid radii shown as *blue* and *orange lines*, respectively) by Minniberger et al. (8). The black line labeled with S_V denotes the position of the selectivity filter. (*d*) Cartoon showing the pore profile (PDB: 7oph).

and are plotted in blue in Fig. 5. The larger elliptical radius b of the PoreAnalyser algorithm is consistently larger than the effective radius r_{eff} of the capsule both for an elliptical CNT 10×50 model system with ratio of elliptical radii $b/a = ½$ depicted in Fig. 5, *a* and *b* and for a GlyR ion channel depicted in Fig. 5, *c* and *d*. The PoreAnalyser algorithm can accurately capture both axes of ellipsoidal probe particles, whereas the HOLE capsule option underestimates the cross-sectional area. The elliptical CNT model system has a volume of $V_{ana} = L\pi ab = 3.1\ k\mathring{A}^3$, where $L = 50\ \mathring{A}$ denotes the length of the carbon nanotube and $2a$ and $2b$ denote the minor and major axis of the elliptical cross-sectional area. The volumes of the model system based on a PoreAnalyser and HOLE profile are $V_{PA} = 2.5\ k\mathring{A}^3$ and $V_{HOLE} = 1.9\ k\mathring{A}^3$, respectively. For the GlyR ion channel, the minima of larger and smaller radii of the PoreAnalyser algorithm coincide. When moving away from the narrowest constrictions, the differences between the major and the minor elliptical axes become more pronounced, possibly reflecting increased deviation away from spherical geometry in the less-restricted region. The pore volume $V_{PA} = 32.4\ k\mathring{A}^3$ obtained from PoreAnalyser is significantly larger than that, $V_{HOLE} = 21.7\ k\mathring{A}^3$, obtained from the HOLE capsule. For both systems, the PoreAnalyser algorithm describes the respective pore more accurately.

CNTs to investigate conductance

To evaluate the accuracy of the PoreAnalyser algorithm when employing ellipsoidal probe particles, a comprehensive analysis was conducted on various CNT model systems. The extent of ellipticity is denoted by two radii; a (longer radius) and b (shorter radius) for each CNT. We enforce the ellipticity through the use of harmonic restraints to maintain the elliptical shape with prescribed ratios b/a of 1, $^3/_4$, and ½ in Fig. 6. We thus first checked to see how well the restraints maintained the desired radius (Fig. 6). Notably, higher standard deviations were observed for CNTs with larger elliptical radii, signifying a pronounced variability when measuring the major axis. The pores identified using spherical probe particles effectively characterized the minor axis, offering a benchmark for comparison. We examined 18 distinct CNTs, each with 6 different areas in the xy plane and 3 varying ratios between the longer and shorter radii (1, $^3/_4$, ½). The HOLE radii of the circular CNTs (corresponding to a ratio of radii of 1) are listed in Table 1. As expected, the HOLE radius, indicative of the shorter radius, aligned well with the minor elliptical axis. However, it was observed that the larger radius was less-accurately maintained (Fig. 6), as might be anticipated from the use of harmonic restraints in this way. Nevertheless, this level of accuracy was deemed acceptable.

Having satisfied ourselves that the model systems were behaving as expected, we then used these CNTs with varying degrees of pore asymmetry as model systems to investigate how pore asymmetry affects ionic conductance. Smart et al. (16) predict the conductance g of a channel based on the pore geometry found by HOLE as

$$R_{HOLE} = \frac{1}{g} = \rho_{bulk} \sum_z \frac{dz}{A_{xy}(z)} \tag{2}$$

where the resistivity ρ_{bulk} of the permeant ions is regarded as a function of the molar ion concentration c. The pore is

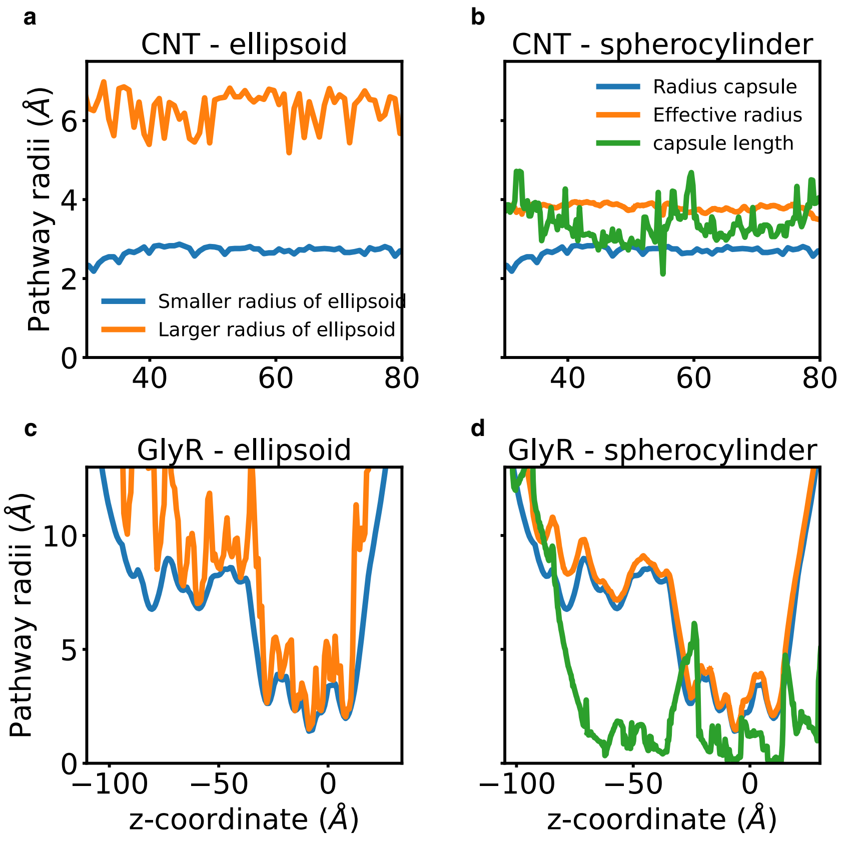

FIGURE 5 Comparison of pathways found with the elliptic probe particle of PoreAnalyser with the CAPSULE option from HOLE. A carbon nanotube profile of a 10 × 50 system with ratio ½ obtained from PoreAnalyser (*a*) and HOLE CAPSULE option (*b*). Profiles obtained from PoreAnalyser (*c*) and HOLE CAPSULE (*d*) for the glycine receptor (PDB: 7TU9).

approximated as a sequence of cylinders with area $A_{xy}(z)$ in the *xy* plane and length *dz*. This model predicts higher resistance *R* (and lower conductance *g*) for longer and narrower pores. However, the model would predict the same conductance for a channel with circular $A_{xy} = \pi r^2$ and for a channel with elliptical area $A_{xy} = \pi ab$ if the cross-sectional area A_{xy} is the same. We measured the conductance and the barrier to permeation for potassium and chloride in four different CNT systems with varying degree of asymmetry. As one would expect, a higher barrier to permeation corresponds to a lower conductance (Fig. 7). Here, the conductance for potassium is lower than for chloride (see also Fig. S2, showing example time series of permeating ion coordinates) and, consistent with this trend, the energetic barrier is higher for potassium than for chloride. We study four different CNT systems with increasing cross-sectional area (CNT 8 × 50 has the lowest cross-sectional area and CNT 14 × 50 the highest), and the conductance increases with increasing cross-sectional area A_{xy} in line with Eq. 2. When we vary the ratio of the elliptical axes while preserving the cross-sectional area A_{xy}, we find that a system with ratio 1, corresponding to a spherical cross-sectional area, has consistently higher conductance values than the same system with ratio ½, corresponding to major axis twice as long as the minor axis. The conductance of chloride with a high standard deviation in the CNT 14 × 50 is an outlier of this trend (Fig. 7 *b*). The energetic barrier decreases for less-elliptical shapes.

We do not observe potassium or chloride conductance events in the repeats of 250 ns in the narrowest CNT system (CNT 8 × 50). The energetic barrier for potassium and chloride to permeate the pore increases with higher eccentricity of the cross-sectional area. The differences are more pronounced for narrower CNTs with smaller cross-sectional areas (Fig. 7). The maximum potential of mean force (PMF) barrier is plotted in Fig. 7, *c* and *d* and the PMF profile along the *z* axis is plotted in Fig. 8, *a* and *b*. Note that we only observe conduction when the PMF barrier is below ~4 kcals/mol. We next calculated the PMFs and estimated the conductance for the ion parameters obtained from Li et al. (39). The barrier for ion permeation and the ion conductance are depicted in Fig. S1. In a recent study we found that ion parameters can influence free energy barriers significantly (37) but, when comparing Figs. 7 and S1, we see a similar trend for energetic barriers and conductances.

We can obtain a measure of the hydration shell along the reaction coordinate by simply reporting the mean and standard deviation of how many water molecules are within 3 Å of the respective ion (Fig. 8, *c* and *d*). The hydration shell of potassium is distorted in the CNT with ratio ½ between the minor and major axes of the elliptical cross-sectional area. The radial distribution function for potassium (Fig. 8 *e*)

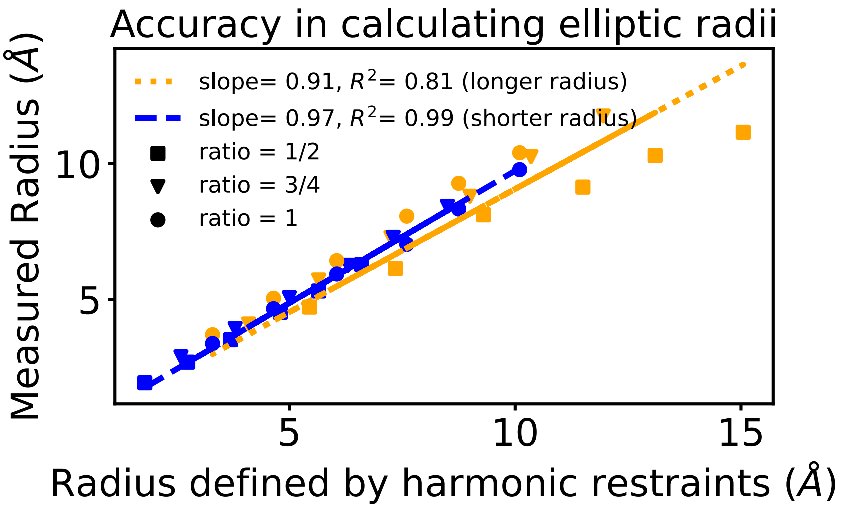

FIGURE 6 Evaluating the accuracy in calculating elliptic radii in 18 different carbon nanotubes (see Table 1) with varying degree of asymmetry. Points are represent as squares, triangles and circles for elliptical ratios ½, ¾, and 1, respectively. It can be seen that the longer radius is less well maintained than the shorter radius, particularly at radii above 9 Å.

and chloride (Fig. 8 *f*) in bulk and in the narrow CNT system reveals that, for potassium, the second peak, corresponding to the second hydration shell, is significantly reduced compared with bulk. Potassium inside a CNT with ratio ½ between the minor and major axes has a smaller radial distribution function compared with potassium inside a spherical CNT (ratio 1).

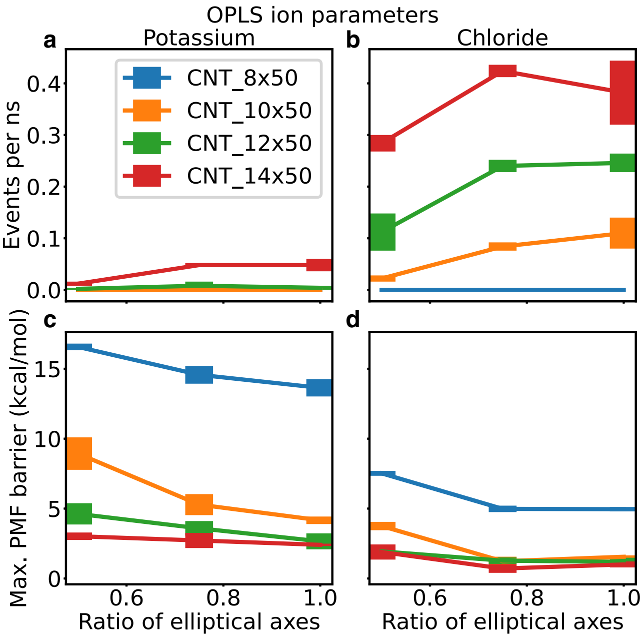

FIGURE 7 Barrier for ion permeation and ion conductance for CNTs as a function of the ratio between the elliptic radii. The conductance is measured as events per ns observed in multiple 250 ns repeats with external potential of 500 mV for (*a*) potassium ions and (*b*) chloride ions. The energetic barrier is the maximum of the potential of mean force (PMF) obtained from umbrella sampling for (*c*) potassium ions and (*d*) chloride ions. The different CNT systems 8 × 50, 10 × 50, 12 × 50, and 14 × 50 (see Table 1 for corresponding HOLE radii) are all 50 Å in length and shown as blue, orange, green and red lines, respectively. Error bars are the standard deviation about the mean of three independent repeats in each case.

Physical model to predict conductance

In the development of a physical model to predict conductance through ion channels, Hille's approach[14] initially considered a cylindrical approximation with length L and cross-sectional area A, allowing the resistance R to be expressed as:

$$R = \frac{1}{g} = \frac{\rho L}{A} \tag{3}$$

where ρ represents bulk resistivity. This simplistic model was subsequently refined (2,40), suggesting a more accurate representation of ion channels as a series of stacked cylinders, where the resistance accumulates. Considering ohmic principles and utilizing the HOLE software, which measures cross-sectional areas $A(z)$ along the channel axis z, the refined resistance model becomes:

$$R_{HOLE} = \frac{1}{g_{HOLE}} = \sum_i \frac{\rho_{bulk}(z_i - z_{i-1})}{\pi \cdot r_i^2} \tag{4}$$

where the resistivity ρ is approximated with bulk property ρ_{bulk} and r_i denotes the radius of a spherical probe particle. However, relying on bulk property resistivity $\rho = {}^1/_\kappa$ becomes problematic, as conductivity κ depends on the diffusion coefficients of ions. The bulk conductivity κ_{bulk} of a KCl solution with concentration c is defined as:

$$\kappa_{bulk} = \frac{q_e^2 c \cdot (D_K + D_{CL})}{k_B T} \tag{5}$$

where q_e is the elementary charge, D_K and D_{CL} are diffusion coefficients of potassium and chloride ions, k_B is the Boltzmann constant, and T is temperature (26). To refine the model for ion channel conductance further, we introduce a conductivity model, expressing the conductivity $\kappa(a_i, b_i)$ as a function of the radii a_i and b_i of ellipsoidal probe particles. For larger radii, the ion movement is relatively unconstrained, resulting in $\kappa(a_i, b_i) \approx \kappa_{bulk}$, while narrower

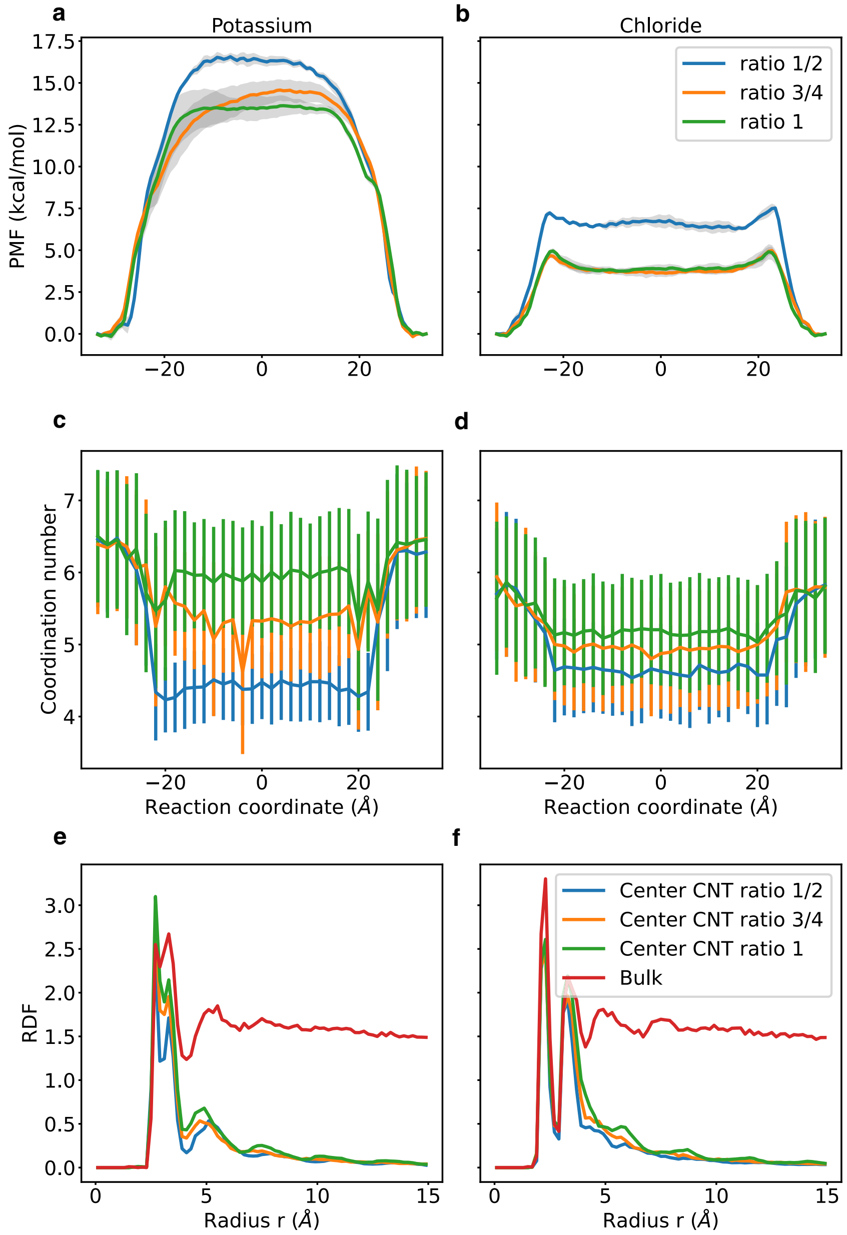

FIGURE 8 PMF and ion hydration shell analysis. PMFs for potassium (*a*) and chloride (*b*) to permeate 8 × 50 CNTs with different degrees of asymmetry. The reaction coordinate is parallel to the *z* axis of the simulation box and the distance to the *z*-coordinate of the center of mass of the CNT. The hydration shell is plotted as a function of the reaction coordinate for potassium (*c*) and chloride (*d*) for a narrow CNT. The radial distribution function (RDF) of potassium (*e*) and chloride (*f*) in bulk and in the center of an 8 × 50 CNT with varying degree of asymmetry. CNT ellipsoid ratios of ½, ¾, and 1 are shown as blue, orange, and green lines, respectively. Bulk values are shown as red lines. Error bars are the standard deviation about the mean of three independent repeats.

constrictions with smaller radii lead to reduced conductivity $\kappa(a_i, b_i) < \kappa_{bulk}$. Hence, we can further adapt the model for channel resistance/conductance based on the PoreAnalyser profile to:

$$R_{PA} = \frac{1}{g_{PF}} = \sum_i \frac{(z_i - z_{i-1})}{\kappa(a_i, b_i) \cdot \pi \cdot a_i \cdot b_i} \quad (6)$$

The conductivity function is represented by a double-sigmoid function with parameters c_1 and c_2, given by:

$$\kappa(a_i, b_i) = \frac{1}{1 + e^{-\frac{a}{c_1} + c_2}} \frac{1}{1 + e^{-\frac{b}{c_1} + c_2}} \kappa_{bulk} \quad (7)$$

We chose a double-sigmoidal function on the grounds of it being one of the simplest nonlinear fitting functions. To fit parameters c_1 and c_2 of the model, training data from 18 CNT systems, encompassing 6 CNTs with distinct cross-sectional areas A_{xy} and three different elliptic radii ratios (a and b), are utilized. Every model system of the training data corresponds to a point in Fig. 9 *a*. In Fig. 9 *b*, the ratio $\kappa(a_i, b_i)/\kappa_{bulk}$ is illustrated as a function of varying radii, keeping one radius constant while the other varies. The conductance of these model systems is calculated based on their mean radii and compared with MD conductance. The conductivity model

$$\kappa(a, b) = \frac{g_{MD}}{\pi \cdot a \cdot b / L} \quad (8)$$

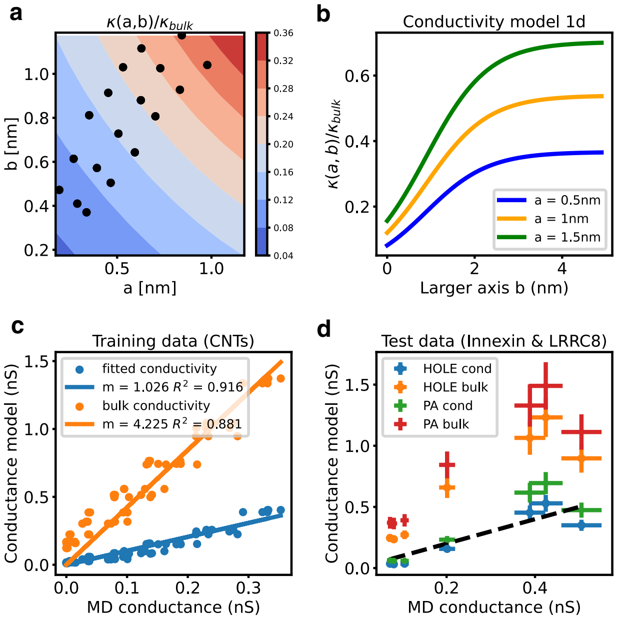

FIGURE 9 Conductivity model for KCl in confined environments characterized by radii a and b. The color surface of the ratio $\kappa(a,b)/\ \kappa_{bulk}$ characterized by the two radii a and b (a). The black dots represent the CNT systems summarized in Table 1 with varying degree of asymmetry. One-dimensional sigmoid function with one radius fixed (b). Comparison of MD conductance (considered to be ground truth) and conductance model using bulk conductivity (*orange*) and conductivity model (*blue*) for the CNT systems summarized in Table 1 (c). Predicting conductance values for biological ion channels (see Table 2) using the bulk conductivity or the conductivity model based on a HOLE (HOLE bulk or HOLE cond) or a PoreAnalyser (PA bulk or PA cond) profile (d). The closer the data points are to the dashed line, the better the model predicts the MD conductance. Error bars in (d) in x (conductance) are standard deviation about the mean derived from three independent repeats. Conductance model estimate is the standard deviation about mean derived from 10 frames of the trajectory.

is employed for fitting, and Fig. 9 *a* depicts the ratio $\kappa(a_i, b_i)/\kappa_{bulk}$ in a two-dimensional plot as a function of both radii, with training data points plotted in the plane spanned by the radii a and b. When utilizing the bulk conductivity κ_{bulk} to predict the conductance of CNTs with Eq. 5, an overestimation of conductance is evident, as depicted in Fig. 9 *c*. However, employing the conductivity model from Eq. 7 yields an improved correlation between MD conductance g_{MD} and the heuristic conductance g_{PA}, resulting in an R^2 value of 0.916.

To further validate our enhanced physical conductance model, we extend the comparison with biological ion channels by embedding various Innexin and LRRC8 structures (see Table 2) in a POPC membrane and measuring MD conductance through simulations with an external potential. In Fig. 9 *d*, we compare MD conductance and the heuristic based on HOLE or PoreAnalyser pathways, both with and without the conductivity model, for the Innexin (41,42) and LRRC8 (43,44) test systems. The use of PoreAnalyser or HOLE pathway profiles in conjunction with bulk conductivity consistently overestimates conductance. Conversely, employing the conductivity model from Eq. 5 with a HOLE pore profile often results in underestimation. The optimal agreement between MD conductance and the heuristic is achieved by using the conductivity model $\kappa(a,b)$ in combination with the PoreAnalyser profile based on ellipsoidal probe particles. The Pearson correlation coefficients of the conductance based on HOLE and PoreAnalyser profiles with the conductivity model are 0.916 and 0.918, respectively. For the INX-6 hemichannel structures (PDB: 6kfg and 6kfh in Table 2) from Burendei et al. (41), we overestimate the conductance and the model based on a HOLE profile overestimates the conductance least. For the other four test systems, the model based on the PoreAnalyser profile performs best. The errors of the MD conductance correspond to one standard deviation of three 250 ns simulations with external potential. To estimate the errors of the conductance model, we analyze the pore pathway for 10 frames from the simulations and calculate the standard deviation.

TABLE 2 Innexin and LRRC8 structures used to test the conductance model

PDB:	Channel	Authors
5h1q	INX-6 gap junction hemichannel	Oshima et al. (42)
6kfg	INX-6 hemichannel in detergent	Burendei et al. (41)
6kfh	INX-6 hemichannel in a nanodisc	Burendei et al. (41)
6m04	homo-hexameric LRRC8D	Nakamura et al. (43)
8b40	homomeric LRRC8C	Rutz et al. (44)
8b41	heteromeric LRRC8A/C	Rutz et al. (44)
8b42	heteromeric LRRC8A/C	Rutz et al. (44)

To further enhance the understanding of the conductance model in Eq. 4 and highlight the contributions of different terms, we present the pore profile as an Innexin system in Fig. 10 *a*. The conductivity along the channel axis is influenced by the radii a and b of the elliptical probe particle, as illustrated by the minima in Fig. 10 *b*. The conductivity $\kappa(r_{HOLE})$ of a HOLE profile is smaller than the conductivity $\kappa(a,b)$ based on the PoreAnalyser profile. Ions, confined in a narrow environment, have a decreased mobility than ions in bulk solution. Calculating resistance based on the cylindrical approximation for each probe particle and plotting it along the channel axis (Fig. 10 *c*), we observe resistance maxima corresponding to the narrow constrictions in Fig. 10 *a*. Substituting the bulk conductivity κ_{bulk} instead of the conductivity model $\kappa(a,b)$ reduces channel resistance, resulting in an overestimation of channel conductance, as demonstrated in Fig. 10 *c*.

CONCLUSIONS

In this study, we focused on capturing pore asymmetry and its influence on conductance and barriers to permeation. Asymmetry can be observed in crystallographic

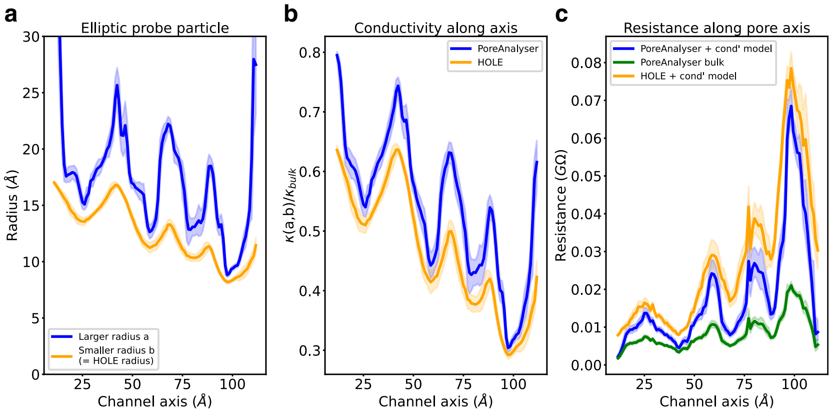

FIGURE 10 Conductivity model for Innexin (PDB: 6kfg, (41)). (*a*) Pore profile described by radii *a* (*blue line*) and *b* (*yellow line*) along the channel axis. (*b*) Conductivity ratio $\kappa(a,b)/\kappa_{bulk}$ along the channel axis as computed by HOLE (*yellow*) and PoreAnalyser (*blue*). (*c*) Resistance along the channel axis employing pore profile based on HOLE (*yellow line*) and PoreAnalyser with (*blue line*) and without (*green line*) the conductivity model (see Eqs. 4 and 6). Errors are one standard deviation derived from an analysis of an ensemble of 10 structures generated from a single MD trajectory.

or cryo-EM structures, which can result from the heterogeneous composition of subunits within the channel complex. While crystal structures of channels with homogenous subunit decomposition exhibit a symmetrical arrangement, the symmetry is broken when these proteins are simulated in more realistic conditions. By incorporating our new features into the PoreAnalyser methodology, we were able to accurately capture and analyze the asymmetry of the pore, shedding light on its functional implications and providing a more comprehensive understanding of ion channel dynamics. The additional complexity of the pore computation adds a slight overhead to the speed of the pathfinding process, which can take approximately 1 min per frame when using 12 CPUs in parallel, but for most use-cases this is a reasonable timing. We demonstrated that consideration of asymmetry allows us to refine a physical conductance model to obtain a heuristic estimate for single-channel conductance. This model allows users to predict single-channel conductance without running costly simulations and can be used as an easy and cheap method for quickly predicting the functional state of new channel structures.

We were also keen to make the package as usable as possible and as such provide an interactive web service (https://poreanalyser.bioch.ox.ac.uk) that allows users to calculate the pore profile of any input structure without the need for installation or downloads. In addition, we have made the source code available on GitHub (https://github.com/bigginlab/PoreAnalyser or https://github.com/DSeiferth/PoreAnalyser) and pip installable, which includes scripts for visualizing results in popular molecular graphics programs such as Chimera, VMD (45), and PyMOL (46).

DATA AND CODE AVAILABILITY

Copies of the source code can be obtained from https://github.com/bigginlab/PoreAnalyser and https://github.com/DSeiferth/PoreAnalyser and include scripts for visualizing results in the molecular graphics programs Chimera, VMD, and PyMOL. The software is pip installable (pip install PoreAnalyser). The software can be used online without any installations via the web service https://poreanalyser.bioch.ox.ac.uk.

SUPPORTING MATERIAL

Supporting material can be found online at https://doi.org/10.1016/j.bpj.2024.07.010.

AUTHOR CONTRIBUTIONS

D.S. and P.C.B. conceived the project. D.S. developed the code. D.S. wrote the first draft and all authors contributed to revising and editing the manuscript.

ACKNOWLEDGMENTS

D.S. is funded by the UKRI-BBSRC Interdisciplinary Bio-science Doctoral Training Partnership (BB/M011224/1). P.C.B. acknowledges support from BBSRC (BB/S001247/1). Computing was supported via the Advanced Research Computing facility, Oxford, and the ARCHER UK National Supercomputing Service and JADE (EP/T022205/1) granted via the High-End Computing Consortium for Biomolecular Simulation, (HECBioSim - http://www.hecbiosim.ac.uk), supported by EPSRC (EP/R029407/1).

DECLARATION OF INTERESTS

The authors declare no competing interests.

REFERENCES

1. Jumper, J., R. Evans, …, D. Hassabis. 2021. Highly accurate protein structure prediction with AlphaFold. *Nature.* 596:583–589. https://doi.org/10.1038/s41586-021-03819-2.
2. Smart, O. S., J. G. Neduvelil, …, M. S. P. Sansom. 1996. Hole: A program for the analysis of the pore dimensions of ion channel structural models. *J. Mol. Graph.* 14:354–376.
3. Stourac, J., O. Vavra, …, D. Bednar. 2019. Caver Web 1.0: identification of tunnels and channels in proteins and analysis of ligand transport. *Nucleic Acids Res.* 47:W414–W422. https://doi.org/10.1093/nar/gkz378.

4. Pravda, L., D. Sehnal, …, M. Otyepka. 2018. MOLEonline: a web-based tool for analyzing channels, tunnels and pores (2018 update). *Nucleic Acids Res.* 46:W368–W373. https://doi.org/10.1093/nar/gky309.

5. Klesse, G., S. Rao, …, S. J. Tucker. 2019. CHAP: a versatile tool for the structural and functional annotation of ion channel pores. *J. Mol. Biol.* 431:3353–3365. https://doi.org/10.1101/527275.

6. Petřek, M., M. Otyepka, …, J. Damborský. 2006. CAVER: a new tool to explore routes from protein clefts, pockets and cavities. *BMC Bioinf.* 7:316. https://doi.org/10.1186/1471-2105-7-316.

7. Chovancova, E., A. Pavelka, …, J. Damborsky. 2012. CAVER 3.0: A tool for the analysis of transport pathways in dynamic protein structures. *PLoS Comput. Biol.* 8:e1002708. https://doi.org/10.1371/journal.pcbi.1002708.

8. Minniberger, S., S. Abdolvand, …, A. J. R. Plested. 2023. Asymmetry and ion selectivity properties of bacterial channel nak mutants derived from ionotropic glutamate receptors. *J. Mol. Biol.* 435:167970.

9. Roy, R. N., K. Hendriks, …, L. Coates. 2021. Structural plasticity of the selectivity filter in a nonselective ion channel. *IUCrJ.* 8:421–430. https://doi.org/10.1107/S205225252100213X.

10. Zhang, Y., P. M. Dijkman, …, M. Kudryashev. 2021. Asymmetric opening of the homopentameric 5-HT3A serotonin receptor in lipid bilayers. *Nat. Commun.* 12:1074. https://doi.org/10.1038/s41467-021-21016-7.

11. Shi, C., Y. He, …, H. Sun. 2018. A single NaK channel conformation is not enough for non-selective ion conduction. *Nat. Commun.* 9:717. https://doi.org/10.1038/s41467-018-03179-y.

12. Lewis, A., V. Kurauskas, …, K. Henzler-Wildman. 2021. Ion-dependent structure, dynamics, and allosteric coupling in a non-selective cation channel. *Nat. Commun.* 12:6225. https://doi.org/10.1038/s41467-021-26538-8.

13. Chugunov, A. O., P. E. Volynsky, …, R. G. Efremov. 2016. Temperature-sensitive gating of TRPV1 channel as probed by atomistic simulations of its trans- and juxtamembrane domains. *Sci. Rep.* 6:33112. https://doi.org/10.1038/srep33112.

14. Gowers, R. J., M. Linke, …, O. Beckstein. 2016. MDAnalysis: A Python package for the rapid analysis of molecular dynamics simulations. *In* Python in Science. S. Benthall and S. Rostrup, eds.

15. Hille, B. 1992. Ionic Channels of Excitable Membranes, 2nd edition. Sinauer Associates Inc, Sunderland, MA.

16. Smart, O. S., J. Breed, …, M. S. P. Sansom. 1997. A novel method for structure-based prediction of ion channel conductance properties. *Biophys. J.* 72:1109–1126. https://www.ncbi.nlm.nih.gov/pmc/articles/PMC1184496/pdf/biophysj00036-0129.pdf.

17. Mejri, A., K. Mazouzi, …, M. Manghi. 2022. Molecular dynamics investigations of ionic conductance at the nanoscale: Role of the water model and geometric parameters. *J. Mol. Liq.* 351:118575. https://doi.org/10.1016/j.molliq.2022.118575.

18. Manghi, M., J. Palmeri, …, V. Jourdain. 2021. Ionic conductance of carbon nanotubes: Confronting literature data with nanofluidic theory. *J. Phys. Chem. C.* 125:22943–22950. https://doi.org/10.1021/acs.jpcc.1c08202.

19. Xing, C., J. Sheng, …, W. Fei. 2021. Research progress in molecular dynamics simulation of CNT and graphene reinforced metal matrix composites. *Oxf. Open Mater. Sci.* 1:itab008. https://doi.org/10.1093/oxfmat/itab008.

20. Rafiee, R., and R. M. Moghadam. 2014. On the modeling of carbon nanotubes: A critical review. *Compos. B Eng.* 56:435–449. https://doi.org/10.1016/j.compositesb.2013.08.037.

21. Seiferth, D., P. C. Biggin, and S. J. Tucker. 2022. When is a hydrophobic gate not a hydrophobic gate? *J. Gen. Physiol.* 154:e202213210. https://doi.org/10.1085/jgp.202213210.

22. Beckstein, O., P. C. Biggin, and M. S. P. Sansom. 2001. A hydrophobic gating mechanism for nanopores. *J. Phys. Chem. B.* 105:12902–12905.

23. Mendonça, B. H. S., D. N. de Freitas, …, A. B. de Oliveira. 2019. Diffusion behaviour of water confined in deformed carbon nanotubes. *Physica A: Stat. Mech. App.* 517:491–498. https://doi.org/10.1016/j.physa.2018.11.042.

24. Mendonça, B. H. S., P. Ternes, …, M. C. Barbosa. 2020. Water diffusion in carbon nanotubes: Interplay between confinement, surface deformation, and temperature. *J. Chem. Phys.* 153:244504. https://doi.org/10.1063/5.0031084.

25. Rezaee, M., M. Namvarpour, …, H. Ghassemi. 2020. Comprehensive study of monatomic fluid flow through elliptical carbon nanotubes. *Phys. Fluids.* 32:092006. https://doi.org/10.1063/5.0020802.

26. Balme, S., F. Picaud, …, J. M. Janot. 2015. Ionic transport through sub-10 nm diameter hydrophobic high-aspect ratio nanopores: experiment, theory and simulation. *Sci. Rep.* 5:10135. https://doi.org/10.1038/srep10135.

27. Mejri, A., N. Arroyo, …, F. Picaud. 2024. Impact of single-walled carbon nanotube functionalization on ion and water molecule transport at the nanoscale. *Nanomaterials.* 14:117.

28. Gibbs, E., E. Klemm, …, S. Chakrapani. 2023. Conformational transitions and allosteric modulation in a heteromeric glycine receptor. *Nat. Commun.* 14:1363. https://doi.org/10.1038/s41467-023-37106-7.

29. Kandt, C., W. L. Ash, and D. Peter Tieleman. 2007. Setting up and running molecular dynamics simulations of membrane proteins. *Methods.* 41:475–488. https://doi.org/10.1016/j.ymeth.2006.08.006.

30. Abraham, M. J., T. Murtola, …, E. Lindahl. 2015. GROMACS: High performance molecular simulations through multi-level parallelism from laptops to supercomputers. *SoftwareX.* 1–2:19–25. https://doi.org/10.1016/j.softx.2015.06.001.

31. Jorgensen, W. L., D. S. Maxwell, and J. Tirado-Rives. 1996. Development and testing of the OPLS all-atom force field on conformational energetics and properties of organic liquids. *J. Am. Chem. Soc.* 118:11225–11236.

32. Berendsen, H. J. C., J. R. Grigera, and T. P. Straatsma. 1987. The missing term in effective pair potentials. *J. Phys. Chem.* 91:6269–6271. https://doi.org/10.1021/j100308a038.

33. Hess, B. 2008. P-lincs: A parallel linear constraint solver for molecular simulation. *J. Chem. Theor. Comput.* 4:116–122. https://doi.org/10.1021/ct700200b.

34. Hess, B., J. Bekker, …, J. G. E. M. Fraaije. 1997. LINCS: A linear constraint solver for molecular simulations. *J. Comput. Chem.* 18:1463–1472.

35. Essman, U., L. Perera, …, L. G. Pedersen. 1995. A smooth particle mesh Ewald method. *J. Chem. Phys.* 103:8577–8593.

36. Darden, T., D. York, and L. Pedersen. 1993. Particle mesh Ewald - an N.log(N) method for Ewald sums in large systems. *J. Chem. Phys.* 98:10089–10092.

37. Seiferth, D., S. J. Tucker, and P. C. Biggin. 2023. Limitations of non-polarizable force fields in describing anion binding poses in non-polar synthetic hosts. *Phys. Chem. Chem. Phys.* 25:17596–17608. https://doi.org/10.1039/D3CP00479A.

38. Hub, J. S., B. L. de Groot, and D. van der Spoel. 2010. g_wham—A Free Weighted Histogram Analysis Implementation Including Robust Error and Autocorrelation Estimates. *J. Chem. Theor. Comput.* 6:3713–3720. https://doi.org/10.1021/ct100494z.

39. Li, P., L. F. Song, and K. M. Merz, Jr. 2015. Systematic parameterization of monovalent ions employing the nonbonded model. *J. Chem. Theor. Comput.* 11:1645–1657. https://doi.org/10.1021/ct500918t.

40. Sansom, M. S. P., and I. D. Kerr. 1995. Transbilayer pores formed by beta-barrels: molecular modeling of pore structures and properties. *Biophys. J.* 69:1334–1343.

41. Burendei, B., R. Shinozaki, …, A. Oshima. 2020. Cryo-EM structures of undocked innexin-6 hemichannels in phospholipids. *Sci. Adv.* 6:eaax3157. https://doi.org/10.1126/sciadv.aax3157.

42. Oshima, A., K. Tani, and Y. Fujiyoshi. 2016. Atomic structure of the innexin-6 gap junction channel determined by cryo-EM. *Nat. Commun.* 7:13681. https://doi.org/10.1038/ncomms13681.

43. Nakamura, R., T. Numata, …, O. Nureki. 2020. Cryo-EM structure of the volume-regulated anion channel LRRC8D isoform identifies features important for substrate permeation. *Commun. Biol.* 3:240. https://doi.org/10.1038/s42003-020-0951-z.

44. Rutz, S., D. Deneka, …, R. Dutzler. 2023. Structure of a volume-regulated heteromeric LRRC8A/C channel. *Nat. Struct. Mol. Biol.* 30:52–61. https://doi.org/10.1038/s41594-022-00899-0.

45. Humphrey, W., A. Dalke, and K. Schulten. 1996. VMD - Visual molecular dynamics. *J. Mol. Graph.* 14:33.

46. The PyMOL Molecular Graphics System DeLano Scientific. San Carlos, CA.

Biophysical Journal, Volume *123*

Supplemental information

Exploring the influence of pore shape on conductance and permeation

David Seiferth and Philip C. Biggin

SUPPLEMENTAL INFORMATION

Exploring the Influence of Pore Shape on Conductance and Permeation

David Seiferth[1,2] and Philip C. Biggin[2]*

[1] Clarendon Laboratory, Department of Physics, University of Oxford, Oxford, OX1 3PU, UK

[2] Structural Bioinformatics and Computational Biochemistry, Department of Biochemistry, University of Oxford, Oxford, OX1 3QU, UK

* To whom correspondence should be addressed:

philip.biggin@bioch.ox.ac.uk

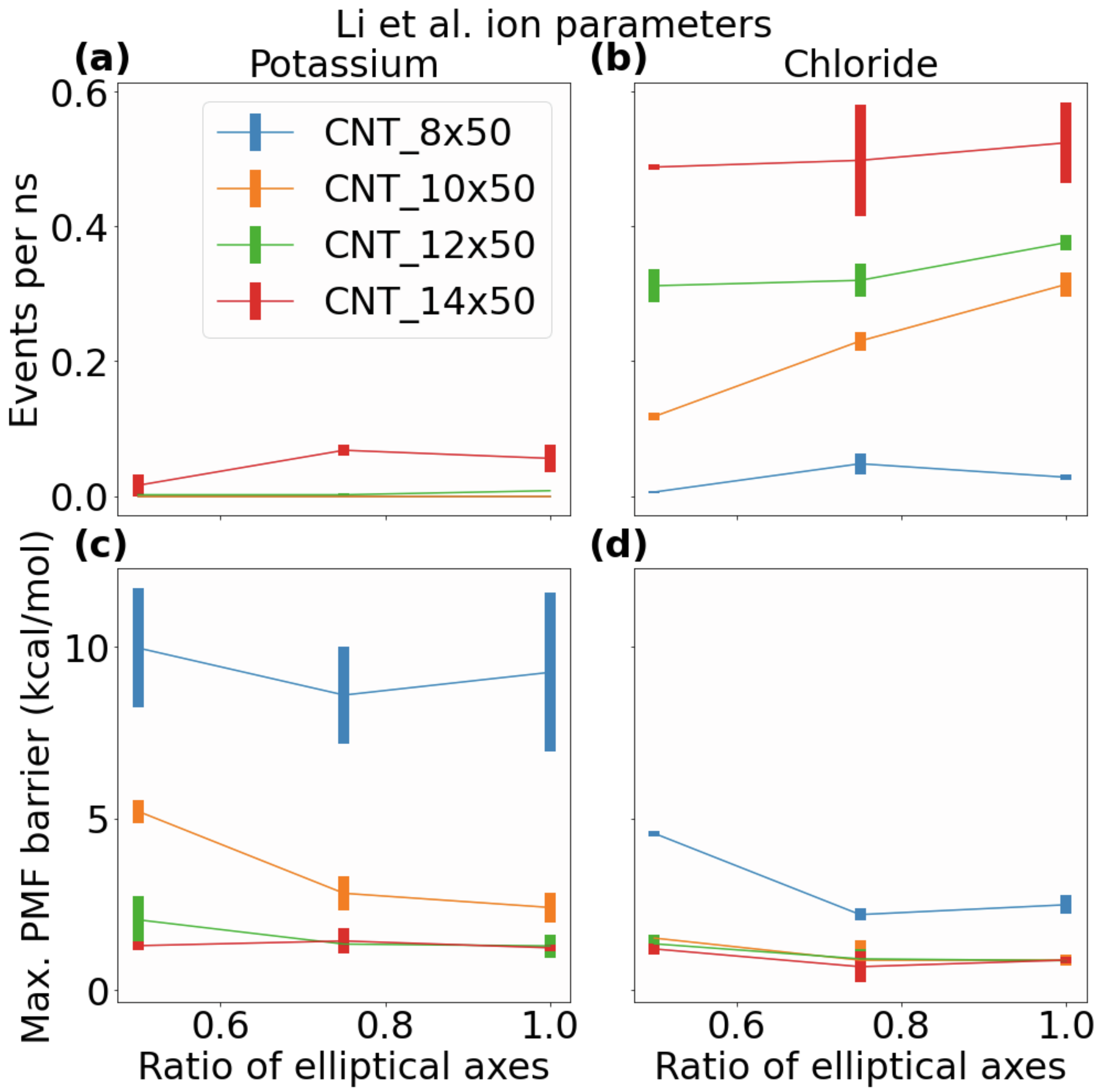

Figure S1. *Barrier for ion permeation and ion conductance for different carbon nanotubes (CNTs) as a function of the ratio between of the elliptic radii using ion parameters from Li et al (2015). The conductance is measured as events per ns observed in multiple 250 ns repeats with external potential of 500 mV for potassium* ***(a)*** *and chloride* ***(b)*** *ions. The energetic barrier is the maximum of the Potential of Mean Force (PMF) obtained from umbrella sampling and is computed for CNTs with potassium* ***(c)*** *and chloride* ***(d)*** *ions. The different CNT systems 8 x 50, 10 x 50, 12 x 50, and 14 x 50 (see Table 1 for corresponding HOLE radii) are all 50 Å in length and shown orange, green and red lines, respectively.*

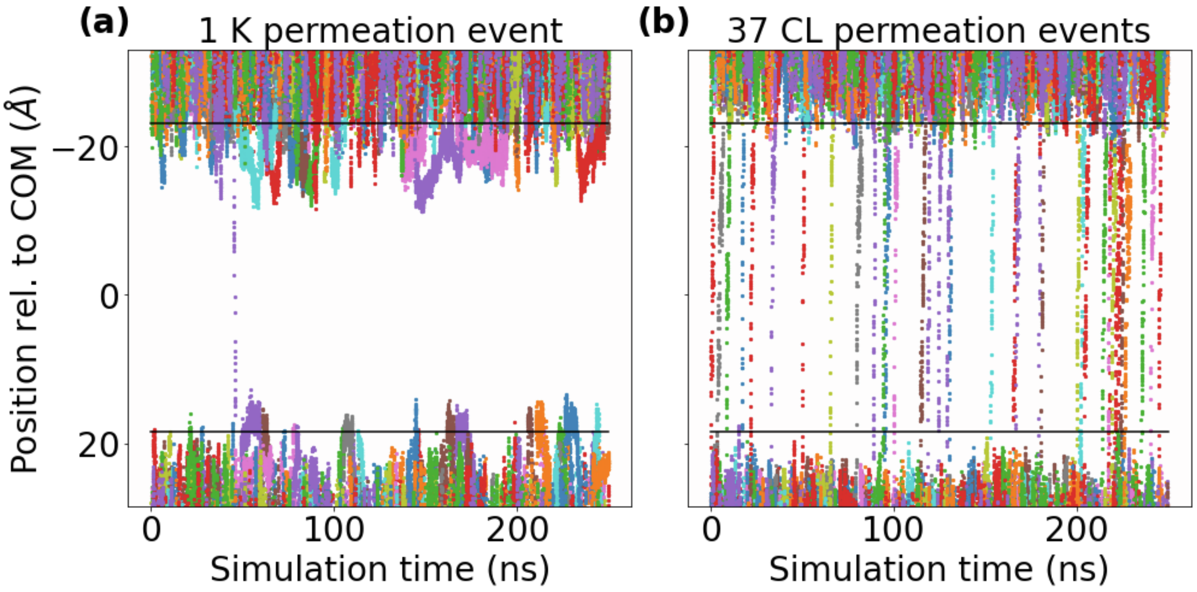

***Figure S2**. Time series of the positions of permeating ions. Every colour corresponds to a different potassium (a) or chloride (b) ion. The black lines represent the position of the phosphate atoms of the POPC bilayer. The z-coordinate of the ions is plotted in a coordinate frame where the centre of mass (COM) of the 12x50 CNT system with ratio ½ is the origin.*
